# In vivo absolute quantification of striatal and extrastriatal D_2/3_ receptors with [^123^I]epidepride SPECT

**DOI:** 10.1186/s13550-020-00650-0

**Published:** 2020-06-16

**Authors:** Stergios Tsartsalis, Benjamin B. Tournier, Philippe Millet

**Affiliations:** 1 Division of Adult Psychiatry, Department of Psychiatry, University Hospitals of Geneva, Chemin du Petit-Bel-Air 2, CH1226, Thônex, Switzerland; 2 Division of Psychiatric Specialties, Department of Psychiatry, University Hospitals of Geneva, Geneva, Switzerland; 3 Department of Psychiatry, University of Geneva, Geneva, Switzerland

**Keywords:** [^123^I]epidepride, D_2/3_ receptor, Dopamine, Molecular imaging, SPECT, Rat

## Abstract

**Background:**

[^123^I]epidepride is a high-affinity radiotracer used in single-photon emission computed tomography (SPECT) imaging of the D_2/3_ receptors. It binds with high affinity to striatal and extrastriatal receptors. Nevertheless, its slow kinetics in the striatum impedes quantification in this region. Thus, an approach that would allow a simultaneous quantification of both striatal and extrastriatal D_2/3_ receptors would be of interest for preclinical and clinical SPECT neuroimaging. We describe a partial saturation protocol that allows us to produce an in vivo Scatchard plot and thus estimate B_avail_ and appK_d_ separately in both striatal and extrastriatal regions, through a single dynamic SPECT session. To validate this approach, a multi-injection protocol is used for the full kinetic modeling of [^123^I]epidepride using a two-tissue compartment, 5-parameter model (2T-5k).

**Methods:**

Eighteen male rats were used. Binding parameters were estimated using the multi-injection protocol. Various simulations were performed to estimate the optimal conditions for the partial saturation protocol, which was applied at the region and voxel level. The results of the partial saturation study were compared to those obtained with the 2T-5k model. To illustrate the interest of the partial saturation approach, we performed a preliminary study of the effect of a chronic, subcutaneous administration of haloperidol (1 mg/kg/day), a D_2_ receptor antagonist, on the B_avail_ of [^123^I]epidepride in the rat striatum.

**Results:**

A series of simulations demonstrated that a mass of 3 ug/kg of unlabeled epidepride allows the formation of an in vivo Scatchard plot. The partial saturation study led to robust estimations of B_avail_ in all brain regions that highly correlated (*r* = 0.99) with the corresponding values from the multi-injection study. A chronic haloperidol treatment resulted in a 17.9% increase in the B_avail_ values in the left Caudate Putamen nucleus (CP) (*p* = 0.07) and a 13.8% increase in the right CP (*p* = 0.12).

**Conclusion:**

A partial saturation method allowed the robust quantification of D_2/3_ receptors in striatal and extrastriatal D_2/3_ receptors with a single-scan approach. This approach may be applied in the mapping of the D_2/3_ receptor in translational biological studies and potentially, in clinical SPECT imaging.

## Background

Molecular imaging of the dopaminergic system with positron emission tomography (PET) and single-photon emission tomography (SPECT) is a powerful tool for the non-invasive study of the living brain in clinical and translational settings. Molecular imaging of the D_2/3_ receptor in particular has provided insight into the pathophysiology of a wide spectrum of neuropsychiatric disorders, ranging from mood and psychotic disorders to addiction and neurodegeneration [1, 2]. Imaging of the D_2/3_ receptor provides information on the receptor density and, after appropriate experimental manipulation, such as the administration of amphetamine or the performance of a neurocognitive test, its interaction with the endogenous ligand, thus allowing an indirect measure of the endogenous dopamine availability [3].

Several D_2/3_-binding radiotracers have been developed and routinely used in research settings. Among them, [^11^C]raclopride and [^123^I]IBZM are perhaps the most widely employed in PET and SPECT imaging of the dopaminergic system, respectively. These radioligands have the disadvantage of a relatively low affinity for the D_2/3_ receptor, limiting their use to imaging of the striatal receptors with a relatively low signal-to-noise binding ratio [4, 5]. For this reason, high-affinity radiotracers have been developed, allowing extrastriatal receptor imaging [5]. Nevertheless, the high affinity of the radiotracer is a serious impediment to the quantification of striatal D_2/3_ receptors. In this region, the kinetics of these radiotracers is particularly slow, meaning that a quantitative approach with standard pharmacokinetic modeling would require an impractical scan duration, both for preclinical and, especially, for clinical imaging [6].

One prominent example of such radiotracers is [^123^I]epidepride [7, 8]. Because of its very high affinity for the D_2/3_ receptor, [^123^I]epidepride is only used for imaging of extrastriatal receptors in human SPECT imaging [9–18]. However, a quantification approach allowing the use of [^123^I]epidepride SPECT for whole-brain imaging would be highly advantageous: on the one hand, its high affinity and thus the high signal-to-noise ratio allow a better sensitivity for the quantification of biological changes compared to lower-affinity radiotracers. On the other hand, compared to PET imaging, SPECT has the advantage of being more accessible to clinical or experimental use, given that it requires no in-house cyclotron (as is the case for PET imaging). ^123^I has a considerably longer half-life (~13 h) than positron-emitting radioisotopes, which is a crucial feature for these high-affinity radiotracers, for which imaging over sufficiently long periods is required to accurately quantify receptor binding. In addition, the development of novel SPECT cameras, in the preclinical and very recently in the clinical level, has led to SPECT imaging with a high spatial resolution, comparable to that PET [19]. Interestingly, the use of ^124^I, a positron-emitting radioisotope has been employed to label epidepride, allowing the use of this radiotracer in PET and providing another argument for the development of epidepride imaging for both SPECT and PET [20].

In this context, a methodology that would allow to quantify [^123/124^I]epidepride in both the striatum and the extrastriatal regions would be of great interest. Given that the main obstacle in [^123^I]epidepride imaging is its very slow kinetics in the striatum, we proposed to employ the partial saturation approach, which, in principle, implies an alteration of the radiotracer’s kinetics. The partial saturation approach has been originally developed for [^11^C]flumazenil imaging of GABA_A_ receptors [21, 22]. In this approach, a dose of an unlabeled ligand is co-injected with the labeled radiotracer. The resulting kinetics of the radiotracer allow an in vivo Scatchard plot to be formed and the B_avail_ and appK_d_ to be separately estimated. This separate estimation of B_avail_ and appK_d_ provides an important advantage: the dissociation of the quantification of the concentration of the receptor from the estimation of appK_d_ potentially removes a confounding effect. Indeed, appK_d_ is directly related to the affinity of the radiotracer for the receptor (1/appK_d_) and may vary as a result of variations in the endogenous ligand of the receptor (i.e., dopamine). This protocol has been recently employed by our group [23] and others [24, 25] for D_2/3_ imaging using [^123^I]IBZM and [^11^C]raclopride in small animals. Overall, this approach has been used to quantify radiotracers with rapid kinetics and this work presents that first application for a radiotracer with slow kinetics, such as [^123^I]epidepride.

In this paper, we firstly perform a full quantification of [^123^I]epidepride kinetics using a multi-injection imaging protocol [23, 26–28], which separately identifies B_avail_ and appK_d_. The results of the multi-injection protocol serve as a “gold-standard” for the validation of the partial saturation approach, which is applied at the region- and at the voxel-level.

## Methods

### Animals and general SPECT scan protocol

18 male Mdr1a (P-glycoprotein) knock-out (KO) rats [29], weighing between 380 and 500 g, were employed in the study. Using this rat strain allowed us to study the kinetics of [^123^I]epidepride independently of the potentially confounding effect of the P-glycoprotein [30–34]. Of these, 3 rats were employed in an in vivo multi-injection SPECT imaging protocol for absolute D_2/3_ receptor quantification (SPECT-MI in Table 1). Four rats were employed in an arterial plasma analysis for the study of plasma kinetics of the radiotracer and the estimation of the free parent radiotracer fraction (TLC in Table 1) [23]. Four rats were employed in a SPECT experiment with a partial D_2/3_ receptor saturation design for the determination of B_avail_ and appK_d_ parameters from an in vivo Scatchard plot (SPECT-PSA in Table 1) [23]. Finally, 7 rats were employed in a preliminary study of the effect of a chronic haloperidol treatment on the B_avail_ and appK_d_ of the D_2/3_ receptors in rats (SPECT-PSA_HAL for haloperidol-treated and SPECT-PSA_CON for control rats in Table 1, see Additional file 1).

**Table 1:** Numerical values of the SPECT and metabolite protocol parameters corresponding to the 18 experiments

Rats	Modality/tracer	Duration (min)	Injection 1 (*T* = 0 min)		Injection 2			Injection 3	
			SA^†^ (GBq/μmol)	J_1_^*^ (MBq/μg)	Time (min)	J_2_^*^ (MBq/μg)	J_2_ (μg)	Time (min)	J_3_ (μg)
1	TLC/[^123^I]epidepride	180	> 1000	89.6/< 0.05	–	–	–	–	–
2	TLC/[^123^I]epidepride	180	> 1000	98.2/< 0.05	–	–	–	–	–
3	TLC/[^123^I]epidepride	180	> 1000	96.9/< 0.05	–	–	–	–	–
4	TLC/[^123^I]epidepride	180	> 1000	77.4/< 0.05	–	–	–	–	–
5	SPECT-MI/[^123^I]epidepride	360	1065.86	95.4/0.043	180	103.4/0.047	1.07	240	214.00
6	SPECT-MI/[^123^I]epidepride	360	967.71	87.4/0.043	180	93.5/0.046	2.35	240	234.50
7	SPECT-MI/[^123^I]epidepride	360	955.45	87.6/0.044	180	97.5/0.049	1.10	240	221.00
8	SPECT-PSA/[^123^I]epidepride	180	19.50	52.7/1.30	–	–	–	–	–
9	SPECT-PSA/[^123^I]epidepride	180	23.74	58.6/1.19	–	–	–	–	–
10	SPECT-PSA/[^123^I]epidepride	180	40.67	113.06/1.34	–	–	–	–	–
11	SPECT-PSA/[^123^I]epidepride	180	29.51	75.24/1.23	–	–	–	–	–
12	SPECT-PSA_CON/[^123^I]epidepride	180	18.85	49.35/1.26	–	–	–	–	–
13	SPECT-PSA_CON/[^123^I]epidepride	180	16.98	43.48/1.23	–	–	–	–	–
14	SPECT-PSA_CON/[^123^I]epidepride	180	22.74	45.65/0.97	–	–	–	–	–
15	SPECT-PSA_CON/[^123^I]epidepride	180	17.10	41.15/1.16	–	–	–	–	–
16	SPECT-PSA_HAL/[^123^I]epidepride	180	18.81	46.09/1.18	–	–	–	–	–
17	SPECT-PSA_HAL/[^123^I]epidepride	180	16.72	40.7/1.17	–	–	–	–	–
18	SPECT-PSA_HAL/[^123^I]epidepride	180	16.95	38.47/1.09	–	–	–	–	–

SPECT scans were performed with a U-SPECT-II camera (MiLabs, Utrecht, Netherlands). In rats that underwent SPECT scans with the multi-injection protocol, two polyethylene catheters (i.d. = 0.58 mm, o.d. = 0.96 mm) were inserted in the left femoral vein and artery for radiotracer administration and blood sampling, respectively. On the other hand, in rats that underwent SPECT scans for the partial saturation protocol, radiotracer injection was performed via a tail vein catheter. SPECT scans were performed under isoflurane anesthesia (3% for induction and 1–2% for maintenance). Body temperature was monitored during the scans and maintained at 37 ± 1 **°**C by means of a thermostatically controlled heating blanket.

SPECT image reconstruction was performed using a pixel-ordered subset expectation maximization (P-OSEM, 0.4-mm voxel size, 4 iterations, 6 subsets) algorithm using MiLabs image reconstruction software. Radioactive decay correction was performed, while correction for attenuation or scatter was not. Following reconstruction, dynamic images from the partial saturation experiment were denoised with factor analysis (FA) using Pixies software (Apteryx, Issy-les-Moulineaux, France) as previously described [23, 27, 35]. FA allows the decomposition of a dynamic signal into a few elementary components, termed factors [27, 28, 36]. In this study, 3 factors were retained and the rest of the signal was discarded as noise [23, 27]. SPECT images were processed with PMOD software v3.7 (PMOD Technologies Ltd, Zurich, Switzerland). Averaged images corresponding to the ten first frames of the acquisition were co-registered to a magnetic resonance imaging (MRI) template integrated in PMOD [37]. Transformation matrices were then applied to dynamic images. Tissue-activity curves (TACs) from the following regions were extracted: caudate-putamen (CP), nucleus accumbens (NAc), ventral tegmental area (VTA), frontal cortex (FC), amygdala (Amy), hypothalamus (Hyp), superior colliculus (SupC), inferior colliculus (InfC), and cerebellum (Cer).

All experimental procedures were approved by the Ethical Committee on Animal Experimentation of the Canton of Geneva, Switzerland. All experimental data, without exception, is available upon request to any of the authors of the manuscript.

### Radiotracer preparation

[^123^I]epidepride was prepared as previously described [7], using a commercially available precursor (ABX, Radenberg, Germany). Radiochemical purity was > 99%. The specific activities in the experiments are shown in Table 1.

### SPECT multi-injection imaging protocol and quantification, arterial plasma analysis, and free parent radiotracer fraction estimation

**Fig. 1:**
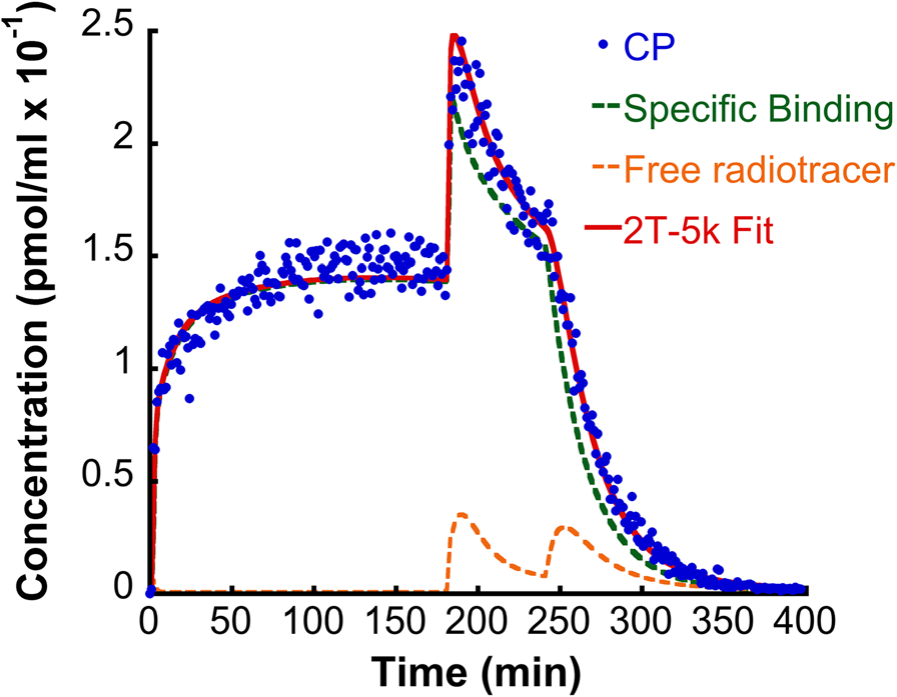
TACs extracted from the CP of one multi-injection dynamic SPECT scan, along with 2T-5k model fit and the decomposition of the radioactive signal into the specific binding and the free radioligand

A multi-injection protocol for full kinetic modeling of [^123^I]epidepride was employed [23, 28, 38]. The scan protocol began with a first injection of the radiotracer at a high-specific activity, followed by a second co-injection of [^123^I]epidepride and the unlabeled compound at 180 min and a third injection of the unlabeled compound alone at 240 min. The overall scan protocol included 360 1-min frames (Fig. 1). The specific activities and radioactive doses of each experiment are presented in Table 1.

The whole multi-injection study TAC data was fitted in with a two-tissue compartment five-parameter model (2T-5k), to estimate K_1_, k_2_, k_on_, k_off_, and B_avail_ as previously described [38–41]. The free, non-metabolized radiotracer fraction in the plasma was used as the input function [42]. During dynamic SPECT acquisitions, 40 arterial blood samples (of 25 μl each) were withdrawn after each radiotracer injection at regular time intervals. Radioactivity was measured in a gamma counting system and expressed in kBq/ml after calibration. To estimate the plasma input function in in vivo SPECT experiments, only whole-blood radioactivity was measured individually and the free, non-metabolized radiotracer fraction was fitted using a model estimated in an independent experiment. Indeed, metabolite correction and plasma protein binding analysis for the estimation of radiotracer plasma input function was performed in an independent group of 4 rats, as previously described [27, 28, 43–46] (the radioactive doses employed in this experiment are presented in Table 1). The analysis was performed in MATLAB software R2019 (Mathworks, USA).

### Simulations and in vivo partial saturation study

#### The partial saturation protocol

A partial saturation protocol was employed to estimate B_avail_ and appK_d_ as recently described for another D_2/3_ radiotracer [23] based on the original description by Delforge et al. [21, 22], as optimized by Wimberley et al. [24, 25]. Briefly, co-injection of a dose of the unlabeled compound at doses that occupy 50–70% of the receptors allows to form a Scatchard plot when the specifically bound fraction of radiotracer (C_S_) is plotted against the ratio of C_S_ to the non-displaceable binding at the same region (C_S_/C_ND_). The intercept to the C_S_ axis provides the B_avail_ and the inverse of the slope the appK_d_.

### Simulation study 1: estimation of the dose of the unlabeled epidepride for the partial saturation study

A first simulation study was performed to estimate the dose of the unlabeled compound that is needed to produce an in vivo Scatchard plot. This simulation study was based on the results of the full quantification from one of the multi-injection experiments. This full-quantification approach allows the complete discrimination of the different components of the radiotracer’s kinetics, namely the free and the specific binding in each VOI. As a consequence, in simulation study 1, single injection experiments using variable doses of unlabeled epidepride were simulated and in vivo Scatchard plots were delineated using the free and the specific binding from the CP (a high-binding) and VTA (a low-binding VOI). This simulation study is particularly important. Indeed, given the potential differences in the in vivo affinity of [^123^I]epidepride for striatal and extrastriatal D_2/3_ receptors (detailed description of the appK_d_ here [47]), as described for other high-affinity D_2/3_ radiotracers [48], the dose-occupancy curves could be different for these two sites. To be able to apply the partial saturation protocol in both striatal and extrastriatal brain region simultaneously, a dose of [^123^I]epidepride which occupies an optimal percentage of the D_2/3_ receptors in both sites to produce an in vivo Scatchard plot is necessary. For this reason, B_avail_ and appK_d_ were estimated and compared to the results of the full quantification using the multi-injection study, to estimate the percent bias in the parameter values as a function of the dose of unlabeled epidepride. The dose of the unlabeled epidepride that allows an optimal estimation of B_avail_ and appK_d_ using the partial saturation method in both striatal and extrastriatal regions was thus determined.

### Estimation of the r parameter for the correction of the cerebellar TAC

In the partial saturation approach, the cerebellar TAC is employed as an approximation of the non-displaceable binding in the target brain VOIs. Given the differences in the concentration of this binding between the Cer and the target VOIs, as well as the presence of a non-negligible specific binding in the Cer with [^123^I]epidepride, the cerebellar TAC has to be corrected. As originally proposed by Delforge et al. [21, 22] and more recently by Wimberley et al. [24], the concentration of the non-displaceable binding in a given brain VOI (C_ND_) is approximated by the corrected concentration of the total (measured) cerebellar binding (C_cer_) using an estimated *r* parameter, so that C_ND_ = *r* × C_cer_.

The estimation of *r* was based on simulation study 1 using the optimal concentration of the unlabeled compound estimated in that study. Radioactivity data corresponding to the time points between the 30th and the 180th minutes from the simulated TACs of the non-displaceable binding in (1) the CP (C_ND-CP_), representing the high-binding, striatal VOIs; (2) the VTA (C_ND-VTA_), representing the low-binding, extrastriatal VOIs; and (3) the total binding in the C_cer_, the reference region were used. The *r* parameter was determined using the ratio *r* = C_ND-CP_/C_cer_ for the striatal VOI and *r* = C_ND-VTA_/C_cer_ for extrastriatal VOIs.

### Simulation study 2: evaluation of the bias induced by the presence of specific binding in the Cer

[^123^I]epidepride has a non-negligible specific binding in the Cer, a region which is used as an approximation of the non-displaceable binding in the whole brain (reference region). The presence of specific binding may induce a bias in the estimation of the quantitative parameters. To evaluate this phenomenon, we employed the results of the full quantification from one of the multi-injection experiments, as in simulation study 1. Various levels of specific binding in the Cer were simulated. A partial saturation experiment, using the optimal dose of unlabeled epidepride as determined in simulation study 1, was simulated. The results of the quantification of B_avail_ and appK_d_ were compared to the results of the full quantification of the multi-injection study and the percent bias in these parameters was estimated as a function of the specific binding in the Cer.

#### In vivo partial saturation study

In vivo partial saturation SPECT scans and image reconstruction were performed in the same conditions as described for the SPECT multi-injection experiments protocol. A single radiotracer injection (containing a dose of unlabeled epidepride determined in the simulation study) was followed by a scan composed of 180 frames of 1 min. No arterial blood sampling took place and the Cer was employed as the reference region. The radioactive doses of each experiment are presented in Table 1. TACs were processed in PMOD (PKIN module), in which the model for the analysis of data from partial saturation experiments, as optimized by Wimberley et al. [24, 25], is implemented.

#### In vivo partial saturation study with a fixed appK_d_ in extrastriatal regions

Given the low binding of [^123^I]epidepride in extrastriatal regions, the estimation of both B_avail_ and appK_d_ may be suboptimal, suffering from high inter-subject variability. For this reason, we performed the same in vivo partial saturation protocol as described in the previous paragraph (“In vivo partial saturation study”) by fixing the appK_d_ value in [22] and thus only fitting B_avail_. This fixed appK_d_ value was determined as the average appK_d_ across the extrastriatal VOIs of the three multi-injection experiments.

### Simulation study 3: impact of fixing the appK_d_ value on the estimation of B_avail_

The appK_d_ parameter may be altered across experimental conditions, notably with respect to the concentration of the endogenous ligand in the vicinity of the receptor under study. As a consequence, if the fixed appK_d_ value in the partial saturation experiments (as in the paragraph “In vivo partial saturation study with a fixed appK_d_ in extrastriatal regions”) differs from the real appK_d_ value, a bias in the estimation of B_avail_ may be induced. To study this phenomenon, we performed a simulation study based on simulation study 1. Using the optimal dose of the unlabeled compound, as determined in that same study, a series of partial saturation experiments was performed, in which varying appK_d_ values were simulated. In vivo Scatchard plots were delineated and B_avail_ was estimated using the same fixed appK_d_ value as determined in “In vivo partial saturation study with a fixed appK_d_ in extrastriatal regions.” The percent bias in the B_avail_ estimates, compared to the simulated B_avail_ values, was calculated.

#### Parametric images of B_avail_ using [^123^I]epidepride

B_avail_ values may also be estimated with the partial saturation approach at the voxel level to produce parametric images [23]. Nevertheless, the partial saturation quantification method that we employed in this study, included in the PKIN module for VOI-wise estimations, has not been included in the PXMOD module of the PMOD software yet, in which parametric estimations are performed. For this reason, to produce parametric images of B_avail_, we applied the partial saturation protocol on a coronal section including the CP and the NAc and another one including the VTA. TACs were extracted from each pixel and processed in PKIN, exactly as described in “In vivo partial saturation study with a fixed appK_d_ in extrastriatal regions”. The resulting pixel-wise B_avail_ values were used to create parametric images in MATLAB.

#### Statistical Analysis

B_avail_ and appK_d_ values resulting from fitting the data of the whole duration of the multi-injection protocol were used as the “gold standard” for comparison of estimations with the partial saturation approach by means of regression analysis. Comparisons of average B_avail_ and appK_d_ values from the multi-injection study and the partial saturation experiments were also performed by means of a two-sample *t* test.

## Results

### Quantitative parameters from the multi-injection study

As described in the “Methods” section describing the multi-injection study protocol, in each dynamic brain SPECT study, only whole-arterial blood radioactivity was measured. The free, non-metabolized radiotracer fraction was estimated by fitting average data from an independent group of four rats in an experiment that was independent from the SPECT scan experiments. The parameters of the bi-exponential function describing the kinetics of the mean percent non-metabolized plasma radiotracer were A_1_ = 0.61, B_1_ = − 0.20, A_2_ = 0.27, B_2_ = − 0.006. Average percentage of radiotracer bound to plasma proteins was 22% (f_1_ = 0.22 ± 0.06). The 2T-5k model provided satisfactory fits to the TACs of the multi-injection study, as shown in Fig. 1. Parameter estimates are provided in Table 2. A non-negligible amount of displaceable binding in Cer is observed (data not shown).

**Table 2:** Mean and standard deviations of binding parameter estimates obtained from 3 rats of the multi-injection study and from 4 rats of the partial saturation study

Multi-injection study													Partial saturation			
VOI	B_avail_	± SD	appK_D_	±SD	Κ_1_	±SD	k_2_	±SD	k_on_	±SD	k_off_	±SD	B_avail_	±SD	appK_D_	±SD
NAc	10.18	1.72	0.15	0.04	0.53	0.14	0.10	0.02	0.26	0.06	0.04	0.00	11.75	2.84	0.27	0.22
CP	24.22	2.62	0.22	0.06	0.69	0.16	0.10	0.04	0.21	0.06	0.04	0.01	21.25	1.25	0.08	0.03
VTA	3.77	1.86	0.34	0.21	0.57	0.07	0.14	0.04	0.28	0.36	0.04	0.02	4.01	1.40	–	–
FC	20.67	22.60	4.50	5.25	0.40	0.15	0.09	0.03	0.01	0.01	0.03	0.01	5.15	2.05	–	–
Amy	2.71	1.53	0.36	0.12	0.38	0.11	0.16	0.10	0.09	0.02	0.03	0.00	2.77	0.72	–	–
Hyp	3.64	1.10	0.37	0.09	0.50	0.21	0.13	0.03	0.15	0.03	0.05	0.01	3.85	0.90	–	–
SupC	3.86	1.65	0.28	0.18	0.70	0.19	0.16	0.04	0.26	0.20	0.05	0.02	3.97	0.93	–	–
InfC	3.03	0.40	0.18	0.02	0.80	0.26	0.17	0.03	0.23	0.02	0.04	0.00	4.46	0.45	–	–
Cer	2.02	1.09	0.65	0.27	0.66	0.18	0.20	0.02	0.04	0.03	0.02	0.01	–	–	–	–

B_avail_ and appK_d_ are in pmol/ml, K_1_ in mL.cm^-3^ .min-^1^, k_2_, k_3_, k_off_, in min^-1^

*NAc* nucleus accumbens, *CP* caudate-putamen, *VTA* ventral tegmental area, *FC* frontal cortex, *Amy* amygdala, *Hyp* hypothalamus, *SupC* superior colliculus, *InfC* inferior colliculus, *Cer* cerebellum

### Simulation studies

#### A simulation study to determine the dose of unlabeled epidepride that is required for the partial saturation protocol

**Fig. 2:**
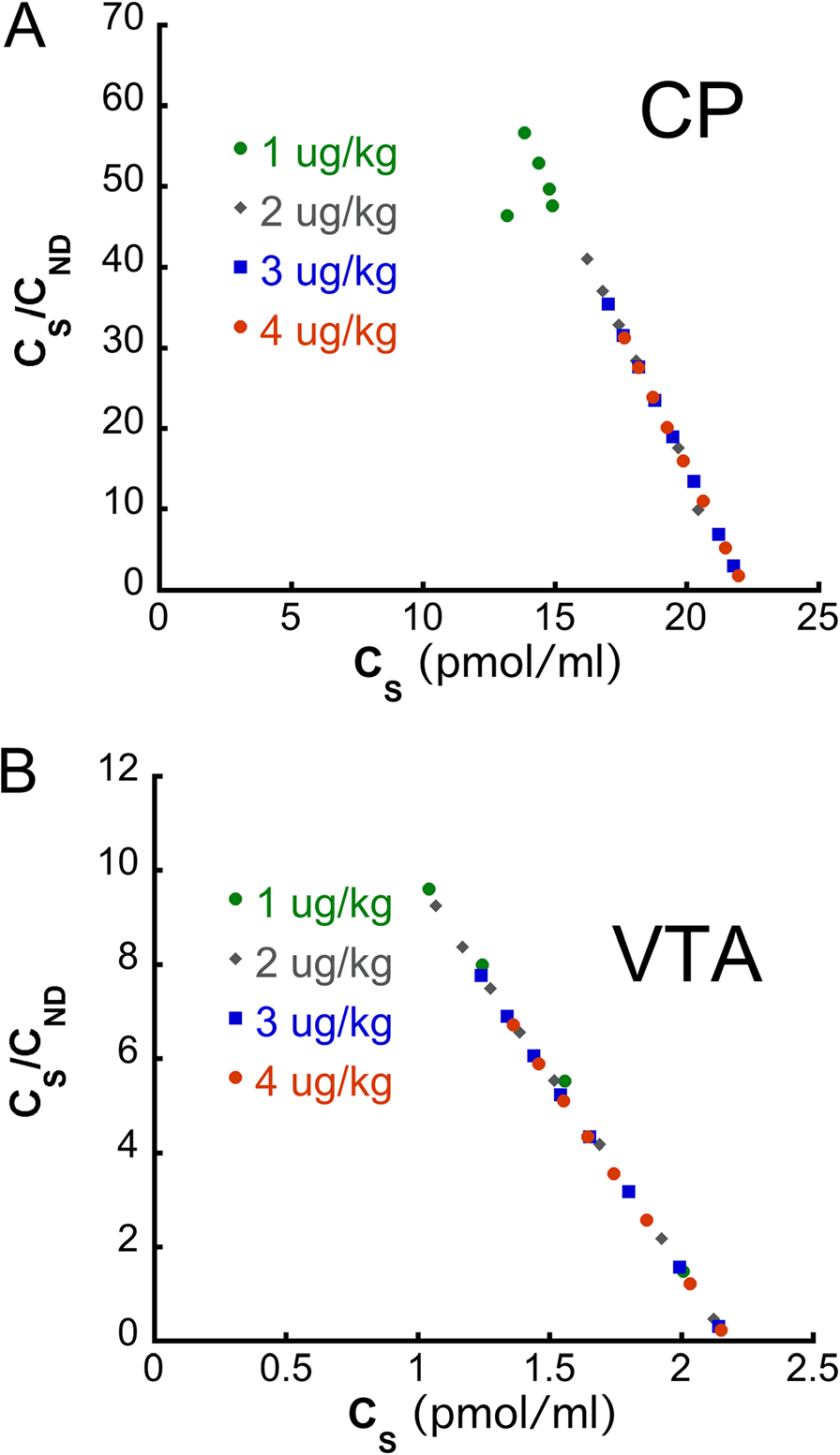
Scatchard plots of the simulation study in the **a** CP (a high-binding VOI) and **b** VTA (a low-binding VOI) for various masses of the injected unlabeled ligand. Note that a dose of the unlabeled ligand of 3 ug/kg or higher produces a Scatchard plot in both VOI. The dose of 3 ug/kg produces a Scatchard plot with the highest range of C_S_/C_ND_ values

As shown in Fig. 2, a dose of unlabeled epidepride of 3 μg/kg or higher produces a Scatchard plot in both high- and low-binding VOI (Fig. 2a,b; Supplemental Fig. 1a, b). We chose to employ this dose of 3 μg/kg, as it produces a Scatchard plot with the highest range of C_S_/C_ND_ values. In this case, the impact of noise in the in vivo experiments should be the lowest.

#### A simulation study to determine the *r* value for the correction of the cerebellar TAC

**Fig. 3:**
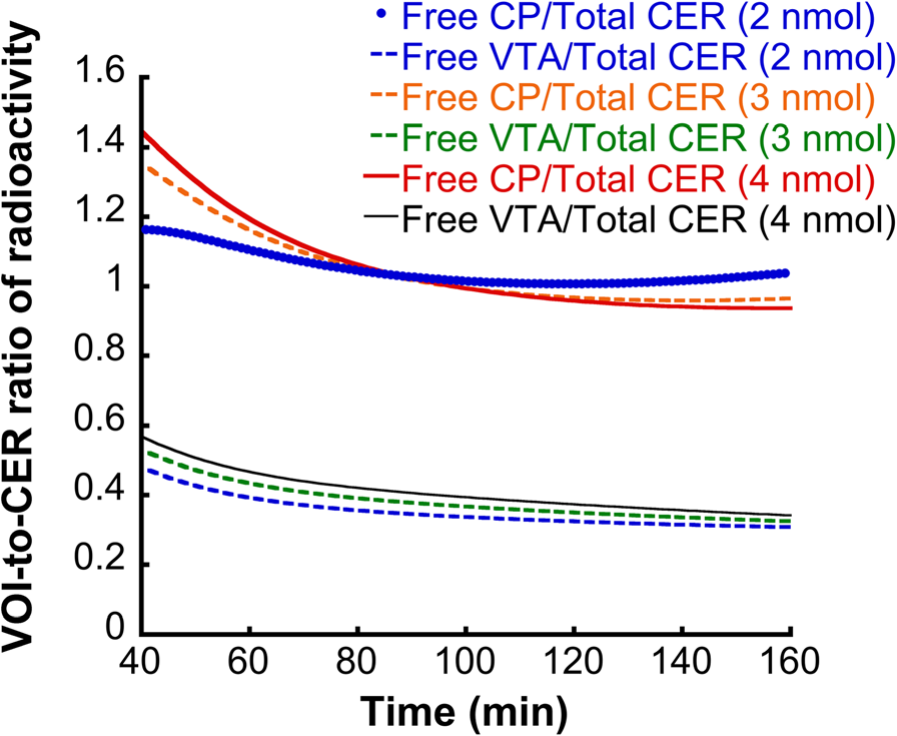
The ratio of radioactivity in the CP (a high-binding VOI) or VTA (a low-binding VOI) to the radioactivity in the Cer (the reference region), as estimated on the basis of a simulated partial saturation experiment, for various masses of injected unlabeled ligand. At time-points beyond 70-min post radiotracer injection, this ratio is around 1 in the CP and 0.4 in the VTA. This ratio is relatively temporally stable at the time points employed in the Scatchard plot quantification with the partial saturation approach

In addition, according to the simulation study (Fig. 3), the *r* value for the correction of the cerebellar TAC was found to be equal to 1 for CP and NAc and equal to 0.4 for the extrastriatal VOI and this value presents an adequate temporal stability in time points beyond 70 min after the injection of the radiotracer, i.e., the time points that are employed in the Scatchard plot. In addition, small variations in the mass of the unlabeled epidepride do not remarkably modify the *r* value.

#### A simulation study to assess the impact of variations in the specific binding in the Cer on B_avail_ and appK_d_ values

**Fig. 4:**
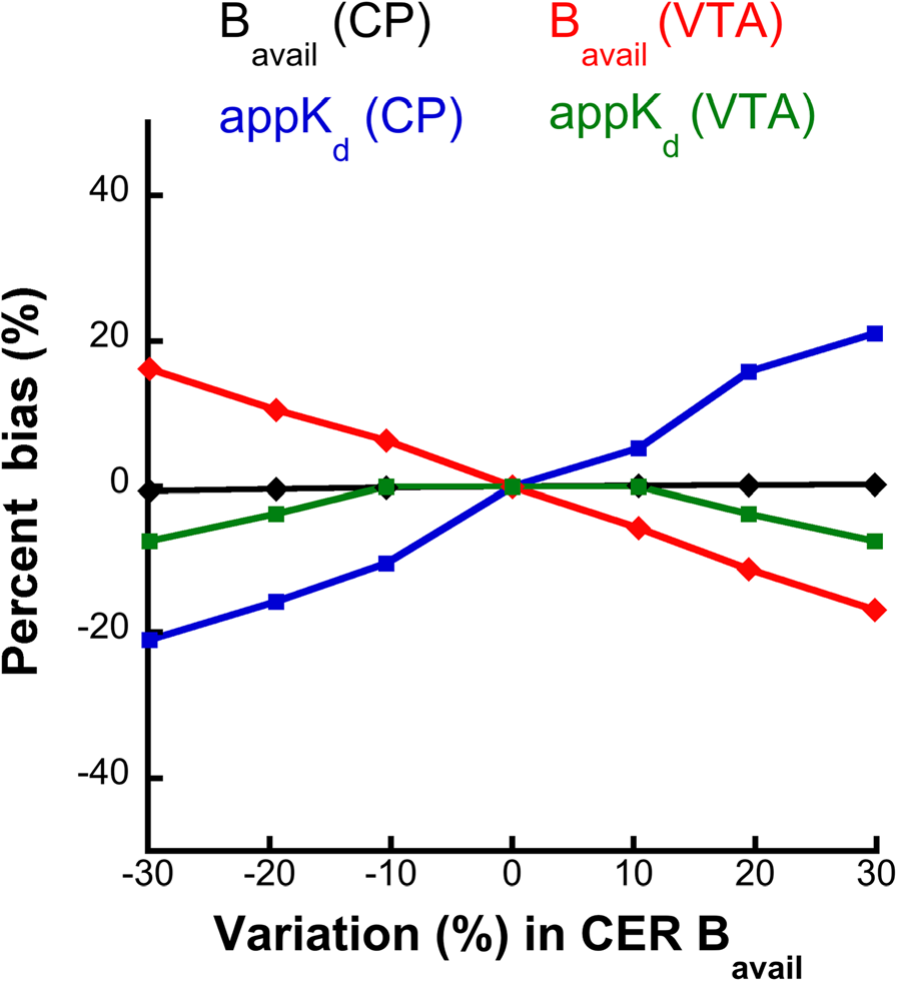
The bias (%), in the B_avail_ and appK_d_ values in the CP and the VTA, induced by a simulated variation (%) in the specific binding in the Cer (reference region), expressed as a variation in the B_avail_ of this VOI. The B_avail_ estimations in the VTA and the appK_d_ estimations in both CP and VTA may be considerably biased in case of a variation in the specific binding in the Cer. The B_avail_ in the CP remains virtually unbiased and independent of any variation in the specific binding in the Cer

The results of the simulation study, in which the impact of varying specific binding levels in the Cer on B_avail_ and appK_d_ are evaluated, are presented in Fig. 4. The B_avail_ values estimated with the partial saturation method from high-binding VOI (the CP) are minimally influenced by this variation in cerebellar specific binding. However, the B_avail_ values from the low-binding VOI (such as the VTA in this simulation) and the appK_d_ values from both high- and low-binding VOI may be biased, depending on the variation in the specific binding in Cer. Indeed, a variation of ± 30% in the specific binding in the Cer will lead to a ± 20% bias in the appK_d_ in the CP, a ± 18% bias in the B_avail_ in the VTA and a ± 7% bias in the appK_d_ in the VTA.

#### Assessing the impact of fixing appK_d_ in extrastriatal B_avail_ value estimations

**Fig. 5:**
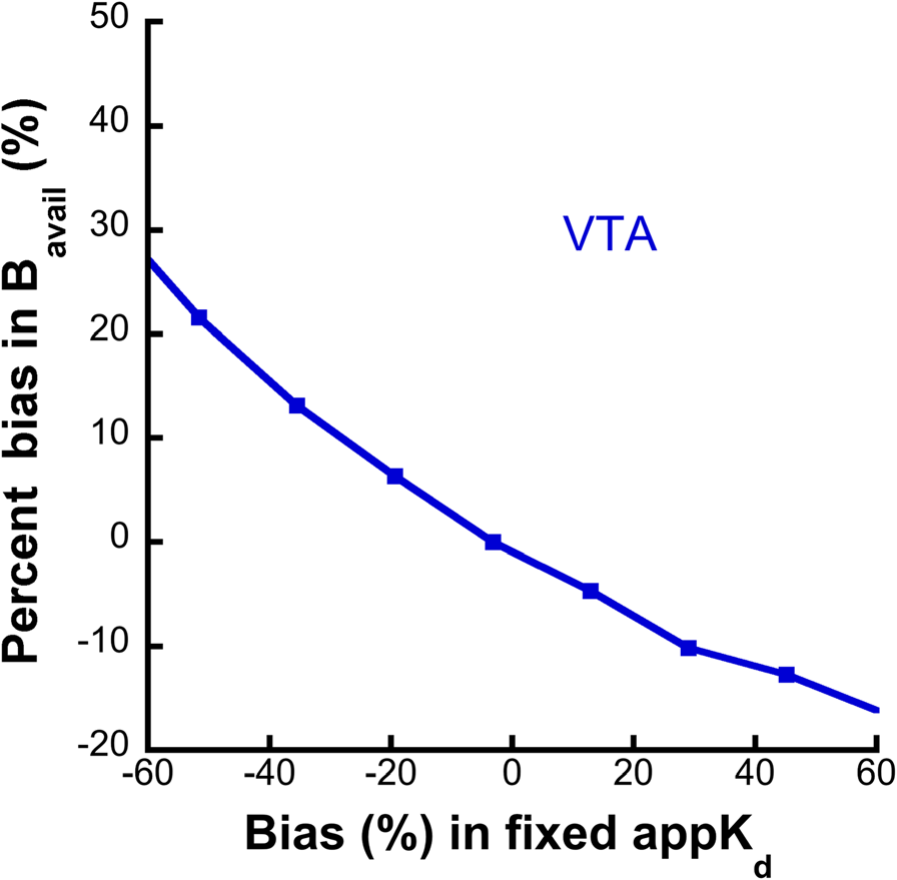
The potential impact of fixing the appK_d_ value in low-binding regions (here the VTA was employed in the simulation study) for the partial saturation quantification. If the “true” appK_d_ of the VOI differs from the fixed value, a bias is introduced in the B_avail_ value estimation. This bias is virtually linearly proportional to the bias in the fixed appK_d_ value

In the last simulation study, the impact of fixing appK_d_ values in extrastriatal B_avail_ value estimations was evaluated. A fixed appK_d_ led to a percent bias in the estimation of the B_avail_ value in the VTA that is almost linear as a function of the percent difference between the “true”-simulated and the fixed appK_d_ (Fig. 5). In detail, a fixed appK_d_ which is higher than the simulated value by 60% leads to an underestimation of the B_avail_ value by 16%. On the contrary, a fixed appK_d_ which is lower than the simulated value by 60% leads to an overestimation of the B_avail_ by 25%.

#### Quantitative parameters from the in vivo partial saturation study at the VOI and pixel level

**Fig. 6:**
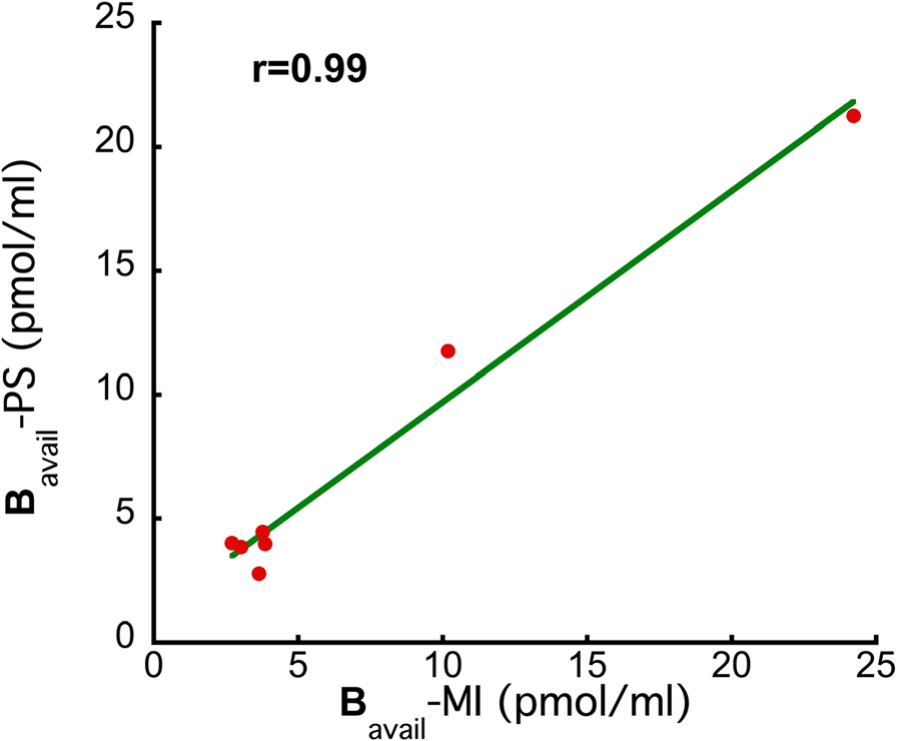
Correlation between the B_avail_ values estimated with the multi-injection approach (horizontal axis) in three rats and the corresponding values estimated with the partial saturation approach (vertical axis)

**Fig. 7:**
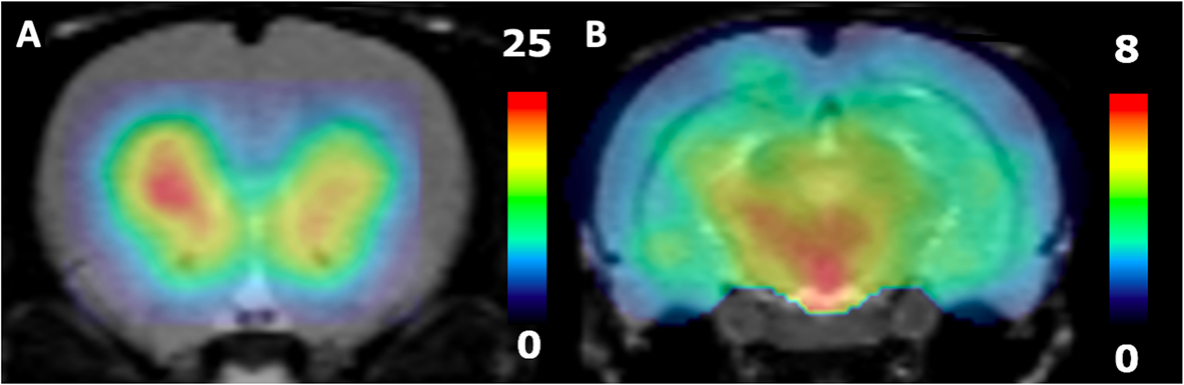
Parametric images of B_avail_ values, estimated with the partial saturation method on a coronal section including **a** the CP and NAc and a section including **b** midbrain structures, notably the VTA. Color bars describe the B_avail_ values in pmol/ml

B_avail_ and appK_d_ values estimated from the in vivo partial saturation experiments are also provided in Table 2. These values are comparable and highly correlated to the corresponding values from the multi-injection experiments, as the linear regression analysis demonstrates (*r* = 0.99, *p* < 0.01 for B_avail_ Fig. 6). In addition, no significant difference in the average B_avail_ or appK_d_ values from the multi-injection and the partial saturation studies was found (*p* > 0.05). The parametric images of B_avail_ in the section including the CP and VTA and in the section including the VTA are shown in Fig. 7.

#### Preliminary study of the effect of chronic haloperidol treatment on the striatal D_2/3_ B_avail_ values

**Fig. 8:**
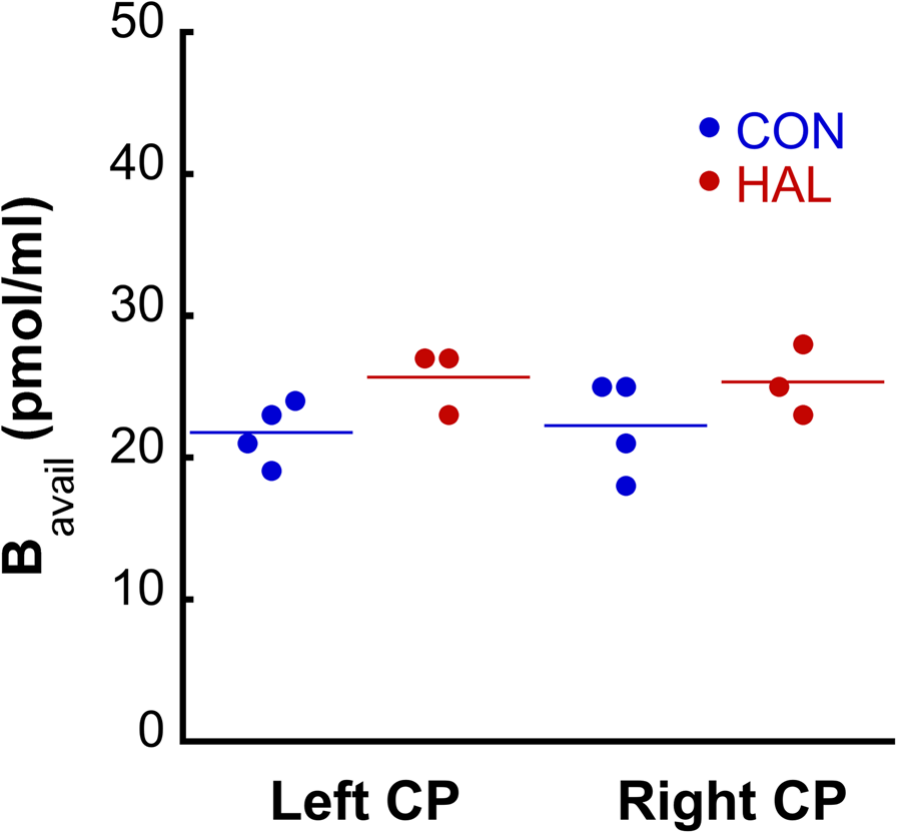
Result of the preliminary study of the effect of a chronic haloperidol treatment on the B_avail_ values in the rat left and right CP

A chronic (28 days) haloperidol treatment (1 mg/kg/day), as described in Additional file 1, resulted in an increase in the B_avail_ values (Fig. 8): 25.67 ± 2.30 pmol/ml in the haloperidol-treated group vs 21.76 ± 2.20 pmol/ml in the vehicle-treated group in the left CP (*p* = 0.07 using two-samples *t* test) and 25.33 ± 2.52 pmol/ml vs 22.25 ± 3.40 pmol/ml in the right CP (*p* = 0.12).

## Discussion

### Validation of B_avail_ and appK_d_ values

In this study, we applied a partial saturation approach in nuclear neuroimaging for the separate quantification of D_2/3_ B_avail_ and appK_d_. The multi-injection approach, the “gold-standard” for the validation of the results of the partial saturation method was also applied in a group of rats. The results of the multi-injection experiment are in accordance with previous studies of absolute D_2/3_ receptor quantification from our group [23], using the same protocol: using [^123^I]IBZM and a multi-injection protocol, B_avail_ values in the CP were found in the range between 19 and 20 pmol/ml. At the level of NAc, the B_avail_ values were found in the range between 13 and 15 pmol/ml, slightly higher than those found in the present study. Considering the extrastriatal VOI, B_avail_ values were roughly between 2 and 4 pmol/ml. In a previous study from our group using [^18^F]fallypride, B_avail_ values were found in the range between 2 and 9 pmol/ml [28]. This difference may be explained by the different delineation of the VOI in the two studies. In the [^18^F]fallypride study, VOI were spherical structures placed in the middle of the corresponding anatomical structure of the brain, whereas in the present study the Schiffer atlas [37] was employed to delineate VOI that correspond to the entire size of the anatomical structure.

For the partial saturation study, we first estimated the *r* parameter that is necessary for the correction of the cerebellar TAC and its use as an index of the non-specific binding of a given VOI in the partial saturation studies. The *r* parameter was estimated using a simulation study in which the total PET signal was decomposed and the non-displaceable binding in striatal and extrastriatal VOI was extracted. The ratio of this non-displaceable binding of the target VOI to the total binding in the Cer gave the *r* parameter. We did not use a pre-saturation in vivo study as described previously from our group [23] and others [24, 25]. This was impossible, because a pre-saturation study would not be able to take the specific binding in the Cer into account in the estimation of *r*. This is because in that case, the specific binding in this region would have been displaced. In addition, we did not estimate the *r* parameter directly by the ratio of the non-displaceable distribution volume ratio the target VOI to the cerebellar VOI (V_ND-target_/V_ND-CER_) because, as shown in Fig. 3, this ratio varies in the first frames of the SPECT scan and it remains relatively stable at later time-points, i.e., those employed in the quantification using the Scatchard plot. As a result, a direct *r* = V_ND-target_/V_ND-CER_ estimate would potentially be biased by the first time-frames, which are not taken into account for the quantification of the B_avail_ and appK_d_. Taking the specific binding in the reference region into account has already been described for the application of the partial saturation approach with [^11^C]flumazenil [22]. In this way, the correction of the TAC of the reference region more accurately represents the ratio of the non-specific binding in the target VOI. Another finding considering the use of the Cer as a reference region in [^123^I]epidepride imaging is illustrated in simulation study 2. The results of this study suggest that should the specific binding in the Cer vary, the B_avail_ values in the low-binding, extrastriatal regions will vary accordingly. B_avail_ values in high-binding regions remain virtually unbiased even with large variations of the specific binding in the Cer. Similarly, appK_d_ estimations in both striatal and extrastriatal regions will vary considerably as a function of the variation in the specific binding in the Cer in a given experimental context (e.g., a brain pathology). [^123^I]epidepride has been employed in multiple studies (discussed in more detail in the following section) of different psychiatric conditions, in which the cerebellar specific binding of the radiotracer is considered unchanged. As demonstrated by Pinborg et al. [49], this issue may be particularly problematic when extrastriatal occupancy of D_2/3_ receptors by pharmaceutical agents is estimated. Possible variations in the specific binding in the Cer have to be taken into account in the design of biological studies using [^123^I]epidepride, and the stability of this specific binding across the experimental conditions has to be verified in order to obtain unbiased results.

The partial saturation approach using [^123^I]epidepride gave highly similar values with previous studies of the D_2/3_ receptor using the same method [23, 24] in the striatum. All B_avail_ values obtained with the partial saturation approach highly correlated with the corresponding B_avail_ values from the multi-injection experiment. However, the appK_d_ values obtained using the partial saturation method in the striatal VOI differ considerably from the corresponding values obtained using the multi-injection approach (Table 2). This is could possibly be due to inter-individual variations in the specific binding in the reference region and in the overall correction of the cerebellar TAC with the *r* parameter. As predicted in the simulation study (Fig. 4), a variation in the specific binding in the Cer induces a considerable bias in the appK_d_ in such high-binding VOI, whereas the B_avail_ values are minimally impacted and this is observed in the estimated parameter values here. Regarding the extrastriatal VOI, the appK_d_ values were fixed in a value obtained from the multi-injection experiment, as attempting to fit both B_avail_ and appK_d_ led to highly variable estimations (data not shown). Fixing appK_d_ in these VOI allowed the estimation of B_avail_ with acceptable variability (Table 2). In this case, as the simulation study suggests (Fig. 5), if the “true” appK_d_ varies, the B_avail_ estimations vary accordingly. This means that, by fixing the appK_d_ parameter value, the B_avail_ becomes a composite parameter which integrates information on both the absolute concentration of the target protein (i.e., the B_avail_ per se) and the affinity of the radiotracer for the target protein (i.e., 1/appK_d_). In this case, in a molecular neuroimaging study involving [^123^I]epidepride, a variation in the B_avail_ value in the extrastriatal VOI in a given experimental condition may be interpreted as either a true elevation of the quantity of the D_2/3_ receptor or a modification of the affinity of the radiotracer for the receptor, which could be linked to alterations in the concentration of dopamine in the synapse. This is essentially similar to the information provided by the binding potential (BP=B_avail_/appK_d_), a composite measure that is most widely employed in molecular neuroimaging [50].

### Potential applications of [^123^I]epidepride imaging using the partial saturation approach

**Fig. 9:**
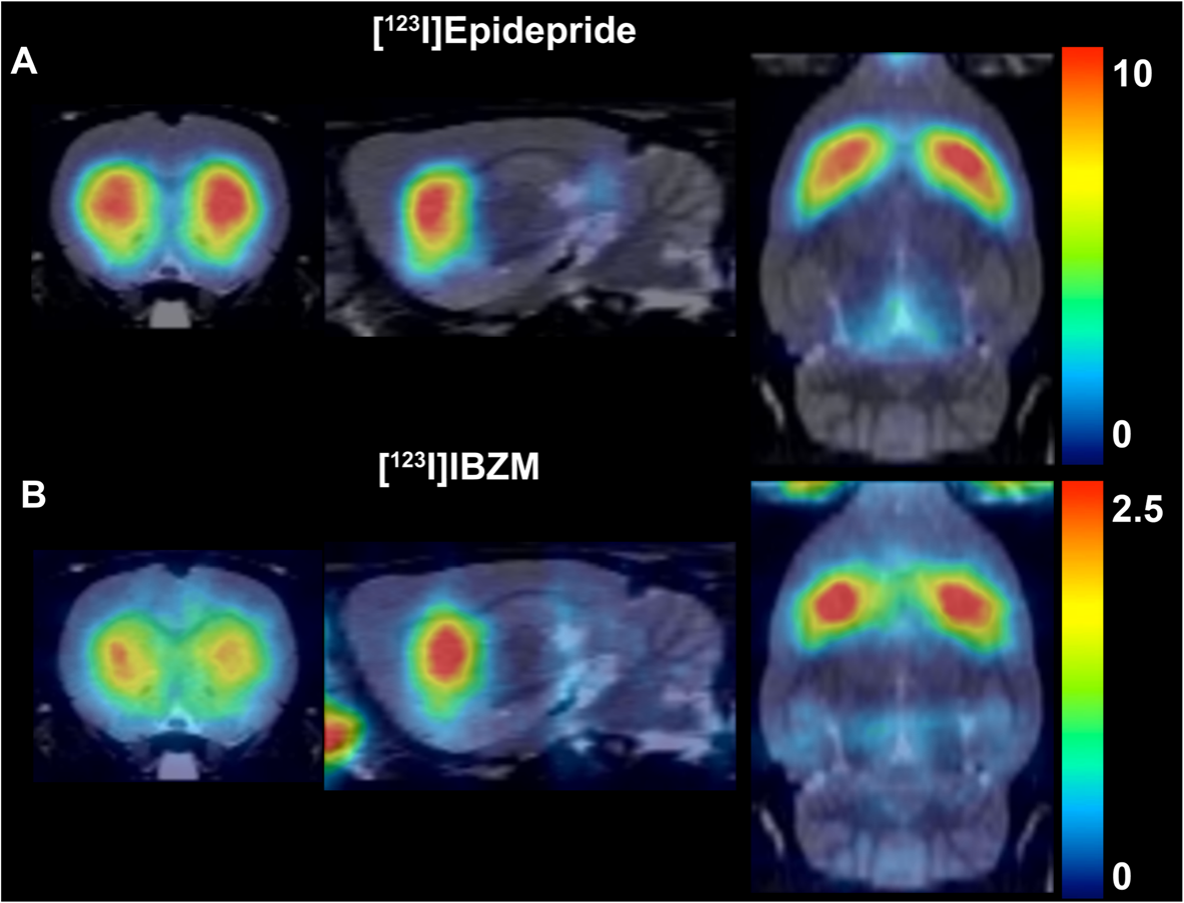
A SPECT image (coronal, sagittal, and axial planes) obtained with **a** [^123^I]epidepride and **b** [^123^I]IBZM. The color scale is in KBq/ml. Note the superior quality of the image obtained from the [^123^I]epidepride experiment in terms of a better anatomical delineation of the striatum and the highest binding of the radiotracer (the color scale of the [^123^I]epidepride image ranges from 0–10 KBq/ml, while the [^123^I]IBZM image ranges have a much lower binding, ranging from 0–2.5 KBq/ml)

The method proposed here is the first approach that allows the quantification of the striatal binding of [^123^I]epidepride. The high affinity of this radiotracer allows to obtain excellent quality images. Figure 9 illustrates the superiority of the quality of a [^123^I]epidepride SPECT image compared to an image obtained with another SPECT D_2/3_ radiotracer, the low-affinity [^123^I]IBZM. The binding of [^123^I]epidepride is considerably higher than the binding of [^123^I]IBZM (note the difference in the range of radioactive concentrations, 0–10 KBq/ml and 0–2.5 KBq/ml, respectively). The high binding of [^123^I]epidepride also allows for an adequate anatomical delineation of the striatal substructures and notably the visual distinction of the NAc from the CP, which is not possible with [^123^I]IBZM.

The quality of the radioactive signal and the robustness of the quantitative parameters that are obtained with the partial saturation approach are supported by the low-standard deviations and the percent coefficients of variation (CV) of the B_avail_ values (Table 2): indeed, the CV of these values obtained with [^123^I]epidepride in the CP is 5.87% and in the NAc is 24.14%. In the extrastriatal regions, CV values range from 10 to 35%. In comparison, the same approach with [^123^I]IBZM yielded B_avail_ values with a CV of 25.43% in the CP and 58% in the NAc in rats [23]. Using the same approach and the low-affinity PET radiotracer [^11^C]raclopride, Wimberley et al. found B_avail_ values with a CV around 40% in mice (as roughly depicted in Fig. 8 of the aforementioned paper) [24]. In rat PET imaging with [^18^F]fallypride [28], CV of BP values estimated with the Logan graphical analysis approach (38.31% in CP and 30% in the NAc and from 38 to 58% in the extrastriatal VOI) were also higher than the CV of B_avail_ values presented here. Overall, the robustness of the quantitative parameters obtained with the partial saturation approach in [^123^I]epidepride SPECT suggests that biological studies using this radiotracer may provide high statistical power compared to other available PET and SPECT imaging modalities of the dopaminergic system.

To illustrate the potential usefulness of the partial saturation approach in biological studies of the dopaminergic system, we performed a preliminary study of the effect of a chronic, high-dose haloperidol treatment [51] on the absolute quantity of D_2/3_ receptors in the striatum. We found an upregulation of the D_2/3_ receptor in the CP, as suggested by previous studies in the literature [52]. Chronic haloperidol treatment led to an increase in the B_avail_ of [^123^I]epidepride of 17.97% in the left CP and of 13.84% in the right CP. Associated *p* values with respect to a comparison by means of a two-sample *t* test were 0.07 and 0.12, respectively, i.e., the differences are not statistically significant using a two-tailed threshold of *p* = 0.05. Indeed, the small sample size (4 rats in the control and 3 in the haloperidol treatment group) suggests that this study was underpowered. However, if a one-tailed significance threshold is considered, the difference in B_avail_ values in the left CP achieves statistical significance. Given the purely illustrative purpose of this experiment, we chose to maintain its preliminary character in the context of the present study. It is for the same reason that we only studied the effect of haloperidol in high-binding VOIs, in which the haloperidol-induced D_2/3_ receptor upregulation has been established in the literature [51, 52], and not in extrastriatal VOIs. Another prominent experimental paradigm where [^123^I]epidepride imaging with the partial saturation protocol is the study of endogenous dopamine alterations in physiological and pathological conditions. It has been experimentally and theoretically established [53] that [^123^I]epidepride binding is not sensitive to “rapid” changes in endogenous dopamine binding in the D_2/3_ receptor, as in the case of amphetamine-induced dopamine release. Nevertheless, using the partial saturation protocol described here, one may gain access to enduring alterations of baseline receptor occupancy by endogenous dopamine in experimental models and pathological conditions, such as in schizophrenia [54, 55].

The high affinity of the binding of [^123^I]epidepride allowed it to be employed in clinical studies of the extrastriatal D_2/3_ receptors in various psychiatric conditions, such as in major depressive disorder [56] and schizophrenia [12, 57, 58]. Using the partial saturation approach presented here, striatal and extrastriatal D_2/3_ receptor binding can be studied simultaneously, which was possible because of the pharmacological properties of [^123^I]epidepride, which allows an optimal percentage of receptors to be saturated in both striatal and extrastriatal regions at the same time, as opposed to other high-affinity D_2/3_ radiotracers (e.g., [^11^C]FLB457 [48]). The interest of dissociating B_avail_ and appK_d_ estimations in PET studies has been discussed extensively in a previous study from our group [23]. The application of the partial saturation protocol in translational [^123^I]epidepride imaging may further potentialize the preclinical studies of the dopaminergic system, well beyond the mesostriatal circuit. The partial saturation protocol described here for preclinical imaging is potentially applicable in clinical PET studies of the dopaminergic system. Indeed, occupying 50–70% of striatal D_2/3_ receptors for the application of the partial saturation approach induces little, if any, adverse effects. Indeed, this level of the blockade is identical to the one induced by antipsychotic agents at therapeutic doses and it is accepted that adverse events appear when the blockade is higher than 80–85% [59]. After the safety of administering pharmacological doses of epidepride is confirmed, the application of this protocol in clinical imaging will be feasible and its impact in the understanding of brain function and disease can be invaluable.

## Conclusions

In conclusion, we propose here, for the first time, a method that allows a simultaneous quantification of striatal and extrastriatal D_2/3_ receptors using [^123^I]epidepride. A cartography of the D_2/3_ receptor in various brain regions is now feasible using this radiotracer. The robustness of the radioactive signal (especially compared to low-affinity D_2/3_ radiotracers) and the stability of the resulting quantitative parameters, which highlight the potential of conducting studies with high statistical power, have been demonstrated. In addition, this approach is relatively simple to apply and already integrated into a commercial image-analysis software. Overall, this study highlights an innovative tool of preclinical brain SPECT imaging in the study of dopamine neurochemistry in physiology and animal models of disease with an approach that is validated for rat SPECT studies but is potentially applicable in clinical brain SPECT.

## Supplementary information

**Additional file 1.** Supplemental Materials and Methods

## Data Availability

All experimental data, without exception, is available upon request to any of the authors of the manuscript.

## Abbreviations

[^123^I]IBZM: [^123^I]-Iodobenzamide
2T-5k: Two-tissue compartment, 5 parameter model
Amy: Amygdala
appK_d_: Dissociation constant
B_avail_: Absolute concentration of the receptor which is accessible for binding of the radioligand in the tissue
BP: Binding potential
C_cer_: Cerebellar binding
Cer: Cerebellum
C_ND_: Non-displaceable binding
CV: Coefficient of variation
CP: Caudate-Putamen
C_s_: Specific binding
D_2/3_: Dopamine receptors 2 and 3
FA: Factor analysis
FC: Frontal cortex
GABA_A_: Gamma aminobutyric acid receptor A
Hyp: Hypothalamus
InfC: Inferior colliculus
KO: Knock-out
MRI: Magnetic resonance imaging
NAc: Nucleus acumbens
PET: Positron emission tomography
P-OSEM: Pixel-based ordered-subset expectation maximization
SPECT: Single-photon emission computed tomography
SPECT-MI: SPECT multi-injection protocol
SPECT-PSA: SPECT partial saturation approach
SPECT-PSA_CON: SPECT partial saturation approach, control group
SPECT-PSA_HAL: SPECT partial saturation approach, haloperidol-treated
SupC: Superior colliculus
TACs: Tissue-activity curves
V_ND_: Non-displaceable volume of distribution
VOI: Volume-of-interest
VTA: Ventral tegmental area

## References

[1] Abi-Dargham A, van de Giessen E, Slifstein M, Kegeles LS, Laruelle M (2009). Baseline and amphetamine-stimulated dopamine activity are related in drug-naive schizophrenic subjects. Biological Psychiatry..

[2] Murnane KS, Howell LL (2011). Neuroimaging and drug taking in primates. Psychopharmacology..

[3] Kugaya A, Fujita M, Innis RB (2000). Applications of SPECT imaging of dopaminergic neurotransmission in neuropsychiatric disorders. Ann Nucl Med.

[4] Laruelle M (2000). Imaging synaptic neurotransmission with in vivo binding competition techniques: a critical review. J Cereb Blood Flow Metab..

[5] de Paulis T (2003). The discovery of epidepride and its analogs as high-affinity radioligands for imaging extrastriatal dopamine D(2) receptors in human brain. Curr Pharm Des.

[6] Ichise M, Fujita M, Seibyl JP, Verhoeff NP, Baldwin RM, Zoghbi SS (1999). Graphical analysis and simplified quantification of striatal and extrastriatal dopamine D2 receptor binding with [123I]epidepride SPECT. J Nucl Med.

[7] Kessler RM, Ansari MS, de Paulis T, Schmidt DE, Clanton JA, Smith HE (1991). High affinity dopamine D2 receptor radioligands. 1. Regional rat brain distribution of iodinated benzamides. J Nucl Med.

[8] Leslie WD, Abrams DN, Greenberg CR, Hobson D (1996). Comparison of iodine-123-epidepride and iodine-123-IBZM for dopamine D2 receptor imaging. J Nucl Med.

[9] Norbak-Emig H, Pinborg LH, Raghava JM, Svarer C, Baare WF, Allerup P, et al. Extrastriatal dopamine D2/3 receptors and cortical grey matter volumes in antipsychotic-naive schizophrenia patients before and after initial antipsychotic treatment. World J Biol Psychiatry. 2016:1–11. 10.1080/15622975.2016.1237042. 10.1080/15622975.2016.1237042 27782768

[10] Nørbak-Emig H, Ebdrup BH, Fagerlund B, Svarer C, Rasmussen H, Friberg L, et al. Frontal D2/3receptor availability in schizophrenia patients before and after their first antipsychotic treatment: relation to cognitive functions and psychopathology. Int J Neuropsychopharmacol. 2016;19:pyw006. doi:10.1093/ijnp/pyw006. 10.1093/ijnp/pyw006 PMC4886673 26819282

[11] Fagerlund B, Pinborg LH, Mortensen EL, Friberg L, Baare WF, Gade A (2013). Relationship of frontal D(2/3) binding potentials to cognition: a study of antipsychotic-naive schizophrenia patients. Int J Neuropsychopharmacol..

[12] Tuppurainen H, Kuikka JT, Viinamaki H, Husso M, Tiihonen J (2009). Dopamine D2/3 receptor binding potential and occupancy in midbrain and temporal cortex by haloperidol, olanzapine and clozapine. Psychiatry Clin Neurosci.

[13] Lehto SM, Kuikka J, Tolmunen T, Hintikka J, Viinamaki H, Vanninen R (2009). Altered hemispheric balance of temporal cortex dopamine D(2/3) receptor binding in major depressive disorder. Psychiatry Res.

[14] Kegeles LS, Slifstein M, Frankle WG, Xu X, Hackett E, Bae SA (2008). Dose-occupancy study of striatal and extrastriatal dopamine D2 receptors by aripiprazole in schizophrenia with PET and [18F]fallypride. Neuropsychopharmacology.

[15] Tuppurainen H, Kuikka JT, Laakso MP, Viinamaki H, Husso M, Tiihonen J (2006). Midbrain dopamine D2/3 receptor binding in schizophrenia. Eur Arch Psychiatry Clin Neurosci.

[16] Fujita M, Seibyl JP, Verhoeff NP, Ichise M, Baldwin RM, Zoghbi SS (1999). Kinetic and equilibrium analyses of [(123)I]epidepride binding to striatal and extrastriatal dopamine D(2) receptors. Synapse..

[17] Varrone A, Fujita M, Verhoeff NP, Zoghbi SS, Baldwin RM, Rajeevan N (2000). Test-retest reproducibility of extrastriatal dopamine D2 receptor imaging with [123I]epidepride SPECT in humans. J Nucl Med.

[18] Fujita M, Verhoeff NP, Varrone A, Zoghbi SS, Baldwin RM, Jatlow PA (2000). Imaging extrastriatal dopamine D(2) receptor occupancy by endogenous dopamine in healthy humans. Eur J Pharmacol.

[19] Meikle SR, Kench P, Kassiou M, Banati RB (2005). Small animal SPECT and its place in the matrix of molecular imaging technologies. Phys Med Biol.

[20] Pandey S, Venugopal A, Kant R, Coleman R, Mukherjee J. (1)(2)(4)I-Epidepride: a PET radiotracer for extended imaging of dopamine D2/D3 receptors. Nucl Med Biol. 2014;41:426-431. doi:10.1016/j.nucmedbio.2014.01.011. 10.1016/j.nucmedbio.2014.01.011 PMC4004701 24602412

[21] Delforge J, Spelle L, Bendriem B, Samson Y, Syrota A (1997). Parametric images of benzodiazepine receptor concentration using a partial-saturation injection. J Cereb Blood Flow Metab..

[22] Delforge J, Spelle L, Bendriem B, Samson Y, Bottlaender M, Papageorgiou S (1996). Quantitation of benzodiazepine receptors in human brain using the partial saturation method. J Nucl Med.

[23] Tsartsalis S, Tournier BB, Aoun K, Habiby S, Pandolfo D, Dimiziani A (2017). A single-scan protocol for absolute D2/3 receptor quantification with [123I]IBZM SPECT. NeuroImage.

[24] Wimberley CJ, Fischer K, Reilhac A, Pichler BJ, Gregoire MC. A data driven method for estimation of B and appK using a single injection protocol with [C]raclopride in the mouse. Neuroimage. 2014. 10.1016/j.neuroimage.2014.05.050. 10.1016/j.neuroimage.2014.05.050 24862069

[25] Wimberley C, Angelis G, Boisson F, Callaghan P, Fischer K, Pichler BJ (2014). Simulation-based optimisation of the PET data processing for Partial Saturation Approach protocols. Neuroimage.

[26] Delforge J, Syrota A, Mazoyer BM (1990). Identifiability analysis and parameter identification of an in vivo ligand-receptor model from PET data. IEEE Trans Biomed Eng.

[27] Tsartsalis S, Moulin-Sallanon M, Dumas N, Tournier BB, Ghezzi C, Charnay Y (2014). Quantification of GABAA receptors in the rat brain with [(123)I]Iomazenil SPECT from factor analysis-denoised images. Nucl Med Biol.

[28] Millet P, Moulin-Sallanon M, Tournier BB, Dumas N, Charnay Y, Ibanez V (2012). Quantification of dopamine D(2/3) receptors in rat brain using factor analysis corrected [18F]Fallypride images. Neuroimage..

[29] Zamek-Gliszczynski MJ, Bedwell DW, Bao JQ, Higgins JW (2012). Characterization of SAGE Mdr1a (P-gp), Bcrp, and Mrp2 knockout rats using loperamide, paclitaxel, sulfasalazine, and carboxydichlorofluorescein pharmacokinetics. Drug Metab Dispos.

[30] Tsartsalis S, Tournier BB, Huynh-Gatz T, Dumas N, Ginovart N, Moulin-Sallanon M (2016). 5-HT2A receptor SPECT imaging with [(1)(2)(3)I]R91150 under P-gp inhibition with tariquidar: more is better?. Nucl Med Biol.

[31] Piel M, Schmitt U, Bausbacher N, Buchholz HG, Grunder G, Hiemke C, et al. Evaluation of P-glycoprotein (abcb1a/b) modulation of [F]fallypride in MicroPET imaging studies. Neuropharmacology. 2013. 10.1016/j.neuropharm.2013.04.062. 10.1016/j.neuropharm.2013.04.062 23994301

[32] Loscher W, Potschka H (2005). Role of drug efflux transporters in the brain for drug disposition and treatment of brain diseases. Progress Neurobiol..

[33] Dumas N, Moulin-Sallanon M, Fender P, Tournier BB, Ginovart N, Charnay Y, et al. In vivo quantification of 5-HT2A brain receptors in Mdr1a KO rats with 123I-R91150 single-photon emission computed tomography. Molecular imaging. 2015;14. 10.2310/7290.2015.00006. 10.2310/7290.2015.00006 26105563

[34] Dumas N, Moulin-Sallanon M, Ginovart N, Tournier BB, Suzanne P, Cailly T, et al. Small-animal single-photon emission computed tomographic imaging of the brain serotoninergic systems in wild-type and mdr1a knockout rats. Molecular imaging. 2014;13. 10.2310/7290.2013.00072. 10.2310/7290.2013.00072 24622810

[35] Tsartsalis S, Tournier BB, Graf CE, Ginovart N, Ibanez V, Millet P (2018). Dynamic image denoising for voxel-wise quantification with statistical parametric mapping in molecular neuroimaging. PLoS One..

[36] Di Paola R, Bazin JP, Aubry F, Aurengo A, Cavailloles F, Herry JY, et al. Handling of dynamic sequences in nuclear medicine. IEEE Trans on Nuclear Science. 1982;NS29:1310-21.

[37] Schiffer WK, Mirrione MM, Biegon A, Alexoff DL, Patel V, Dewey SL (2006). Serial microPET measures of the metabolic reaction to a microdialysis probe implant. J Neurosci Methods..

[38] Millet P, Graf C, Moulin M, Ibanez V (2006). SPECT quantification of benzodiazepine receptor concentration using a dual-ligand approach. J Nucl Med.

[39] Millet P, Delforge J, Mauguiere F, Pappata S, Cinotti L, Frouin V (1995). Parameter and index images of benzodiazepine receptor concentration in the brain. J Nucl Med.

[40] Ginovart N, Wilson AA, Meyer JH, Hussey D, Houle S (2001). Positron emission tomography quantification of [(11)C]-DASB binding to the human serotonin transporter: modeling strategies. J Cereb Blood Flow Metab..

[41] Millet P, Ibanez V, Delforge J, Pappata S, Guimon J (2000). Wavelet analysis of dynamic PET data: application to the parametric imaging of benzodiazepine receptor concentration. Neuroimage..

[42] Delforge J, Bottlaender M, Loc'h C, Guenther I, Fuseau C, Bendriem B (1999). Quantitation of extrastriatal D2 receptors using a very high-affinity ligand (FLB 457) and the multi-injection approach. J Cereb Blood Flow Metab..

[43] Tsartsalis S, Dumas N, Tournier BB, Pham T, Moulin-Sallanon M, Gregoire MC (2015). SPECT imaging of glioma with radioiodinated CLINDE: evidence from a mouse GL26 glioma model. EJNMMI Res..

[44] Millet P, Moulin M, Bartoli A, Del Guerra A, Ginovart N, Lemoucheux L (2008). In vivo quantification of 5-HT1A-[18F]MPPF interactions in rats using the YAP-(S)PET scanner and a beta-microprobe. Neuroimage..

[45] Mintun MA, Raichle ME, Kilbourn MR, Wooten GF, Welch MJ (1984). A quantitative model for the in vivo assessment of drug binding sites with positron emission tomography. Ann Neurol..

[46] Gandelman MS, Baldwin RM, Zoghbi SS, Zea-Ponce Y, Innis RB (1994). Evaluation of ultrafiltration for the free-fraction determination of single photon emission computed tomography (SPECT) radiotracers: beta-CIT, IBF, and iomazenil. J Pharm Sci.

[47] Delforge J, Syrota A, Bendriem B (1996). Concept of reaction volume in the in vivo ligand-receptor model. J Nucl Med.

[48] Delforge J, Bottlaender M, Loc'h C, Dolle F, Syrota A (2001). Parametric images of the extrastriatal D2 receptor density obtained using a high-affinity ligand (FLB 457) and a double-saturation method. J Cereb Blood Flow Metab..

[49] Pinborg LH, Videbaek C, Ziebell M, Mackeprang T, Friberg L, Rasmussen H (2007). [123I]Epidepride binding to cerebellar dopamine D2/D3 receptors is displaceable: implications for the use of cerebellum as a reference region. NeuroImage..

[50] Innis RB, Cunningham VJ, Delforge J, Fujita M, Gjedde A, Gunn RN (2007). Consensus nomenclature for in vivo imaging of reversibly binding radioligands. J Cereb Blood Flow Metab..

[51] Turrone P, Remington G, Kapur S, Nobrega JN (2003). Differential effects of within-day continuous vs. transient dopamine D2 receptor occupancy in the development of vacuous chewing movements (VCMs) in rats. Neuropsychopharmacology.

[52] Ginovart N, Wilson AA, Hussey D, Houle S, Kapur S (2008). D2-Receptor upregulation is dependent upon temporal course of D2-occupancy: a longitudinal [11C]-raclopride PET study in cats. Neuropsychopharmacology.

[53] Morris ED, Yoder KK (2007). Positron emission tomography displacement sensitivity: predicting binding potential change for positron emission tomography tracers based on their kinetic characteristics. J Cereb Blood Flow Metab..

[54] Caravaggio F, Iwata Y, Kim J, Shah P, Gerretsen P, Remington G, et al. What proportion of striatal D2 receptors are occupied by endogenous dopamine at baseline? A meta-analysis with implications for understanding antipsychotic occupancy. Neuropharmacology. 2019. 10.1016/j.neuropharm.2019.03.034. 10.1016/j.neuropharm.2019.03.034 30940535

[55] Kegeles LS, Abi-Dargham A, Frankle WG, Gil R, Cooper TB, Slifstein M (2010). Increased synaptic dopamine function in associative regions of the striatum in schizophrenia. Arch Gen Psychiatry.

[56] Lehto SM, Kuikka J, Tolmunen T, Hintikka J, Viinamaki H, Vanninen R (2008). Temporal cortex dopamine D2/3 receptor binding in major depression. Psychiatry Clin Neurosci.

[57] Tuppurainen H, Kuikka J, Viinamaki H, Husso-Saastamoinen M, Bergstrom K, Tiihonen J (2003). Extrastriatal dopamine D 2/3 receptor density and distribution in drug-naive schizophrenic patients. Molecular Psychiatry..

[58] Glenthoj BY, Mackeprang T, Svarer C, Rasmussen H, Pinborg LH, Friberg L (2006). Frontal dopamine D(2/3) receptor binding in drug-naive first-episode schizophrenic patients correlates with positive psychotic symptoms and gender. Biological psychiatry..

[59] Ginovart N, Kapur S. Role of dopamine D(2) receptors for antipsychotic activity. Handb Exp Pharmacol. 2012:27–52. 10.1007/978-3-642-25761-2_2. 10.1007/978-3-642-25761-2_2 23129327

