# Supplementary material for: In vivo absolute quantification of striatal and extrastriatal D2/3 receptors with [123I]epidepride SPECT
Source: EJNMMI Res. 2020 Jun 16;10:66. doi: 10.1186/s13550-020-00650-0 (PMC7297889; doi:10.1186/s13550-020-00650-0)
Supplement: Supplementary file 1 — Additional file 1. Supplemental Materials and Methods [file 13550_2020_650_MOESM1_ESM.zip › Supplement_ESM.docx]

**Supplement**

***Supplemental Materials and Methods***

*A preliminary study of the effect of a chronic haloperidol treatment on D_2/3_ B_avail_ and appK_d_*

To demonstrate the potential of the partial saturation method proposed here, we performed a preliminary, proof-of-concept study of the effect of a chronic haloperidol treatment on the B_avail_ and the appK_d_ of D_2/3_ receptors in the rat brain. For this purpose, two groups of rats were treated with either haloperidol (1 mg/kg/day, n=3) or vehicle (n=4) for 28 days. A [^123^I]epidepride SPECT scan using the partial saturation protocol was performed 7 days (a sufficient period for the complete elimination of haloperidol) after the end of the treatment period to assess neurochemical changes with respect to the D_2/3_ receptor. The radioactivity doses that were employed are presented in Table 1 (SPECT-PSA_HAL for haloperidol-treated and SPECT-PSA_CON for control rats). Statistical analysis of the difference in the quantitative parameter values between the groups was performed by means of a two-sample t test.

*Osmotic minipump preparation and surgery*

Haloperidol was purchased from Sigma-Aldrich (Buchs, Switzerland). It was dissolved in dimethyl-sulfoxide (DMSO) 50% v/v in water. Osmotic pumps (Alzet, Durect, Cupertino, CA, USA) were employed to continuously deliver the treatment at a dose of 1 mg/kg/day over 28 days. For the pump implantation, rats were anesthetized with isoflurane (3% for induction and 1-2 % for maintenance). Body temperature was monitored during the scans and maintained at 37±1 **°**C by means of a thermostatically controlled heating blanket. An incision was performed in the lateral abdominal wall and a pump was subcutaneously implanted. Subcutaneous analgesia (0.02 mg/kg/8h sc; Temgesic, Reckitt Benckiser Pharmaceuticals Inc) was administered right before surgery and was continued *per os* in the drinking water for 48h. For the removal of the osmotic pumps, the same surgical procedure was performed after the end of the treatment period (28 days).

***Supplemental Figure Legends***

Supplemental Figure 1 Representative Scatchard plots in (a) the CP and (b) the VTA from one experiment. This figure shows that under a dose of unlabeled Epidepride of 3 ug/kg, Scatchard plots are formed in both striatal and extrastriatal VOI.
