# Supplementary figures and images for: In vivo absolute quantification of striatal and extrastriatal D2/3 receptors with [123I]epidepride SPECT
Source: EJNMMI Res. 2020 Jun 16;10:66. doi: 10.1186/s13550-020-00650-0 (PMC7297889; doi:10.1186/s13550-020-00650-0)

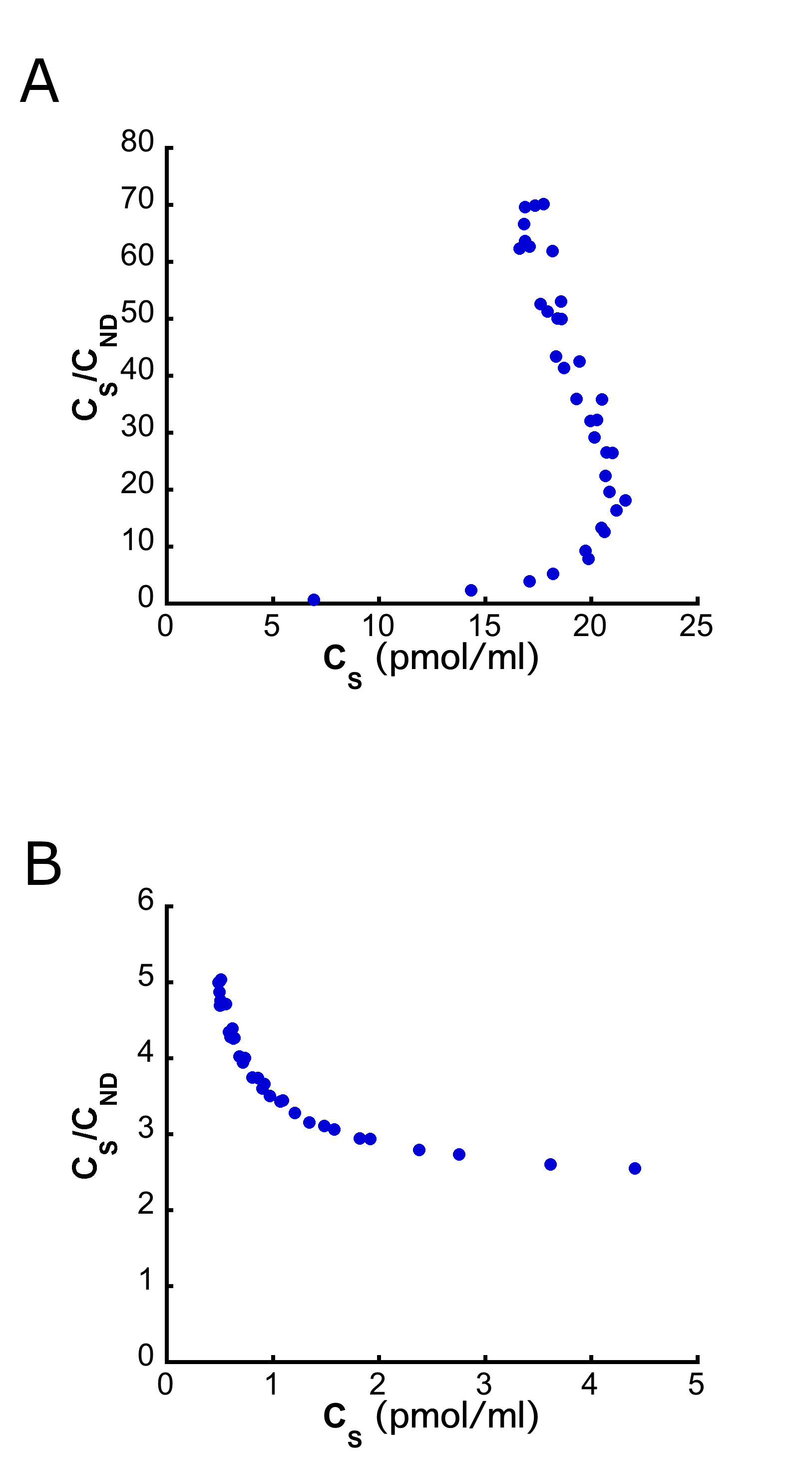

Supplement: Supplementary file 1 — Additional file 1. Supplemental Materials and Methods [file 13550_2020_650_MOESM1_ESM.zip › Supplemental Figure 1_ESM.tif]
